# Natural history of patients with Leber hereditary optic neuropathy—results from the REALITY study

**DOI:** 10.1038/s41433-021-01535-9

**Published:** 2021-04-28

**Authors:** Patrick Yu-Wai-Man, Nancy J. Newman, Valerio Carelli, Chiara La Morgia, Valérie Biousse, Francesco M. Bandello, Catherine Vignal Clermont, Lorena Castillo Campillo, Stephanie Leruez, Mark L. Moster, Dean M. Cestari, Rod Foroozan, Alfredo Sadun, Rustum Karanjia, Neringa Jurkute, Laure Blouin, Magali Taiel, José-Alain Sahel, for the LHON REALITY Study Group, Rima Hussain, Rasha Jorany, Priyansha Sheel, Lindreth DuBois, Michele Carbonelli, Lidia Di Vito, Martina Romagnoli, Adam A. DeBusk, Maria Massini, Rabih Hage, Gad Heilweil, Irena Tsui, Virginia Garcia, Antonio Morilla, Piero Barboni, Maria Lucia Cascavilla, Marco Battista, Francesca Calcagno, Adelaide Pina

**Affiliations:** 1 Cambridge Centre for Brain Repair and MRC Mitochondrial Biology Unit, Department of Clinical Neurosciences, University of Cambridge, Cambridge, UK; 2 Cambridge Eye Unit, Addenbrooke’s Hospital, Cambridge University Hospitals, Cambridge, UK; 3 Moorfields Eye Hospital, London, UK; 4 UCL Institute of Ophthalmology, University College London, London, UK; 5 Departments of Ophthalmology, Neurology and Neurological Surgery, Emory University School of Medicine, Atlanta, GA USA; 6 IRCCS Istituto delle Scienze Neurologiche di Bologna, UOC Clinica Neurologica, Bologna, Italy; 7 Unit of Neurology, Department of Biomedical and Neuromotor Sciences (DIBINEM), University of Bologna, Bologna, Italy; 8 Department of Ophthalmology, Vita-Salute San Raffaele University, Milan, Italy; 9 IRCCS San Raffaele Scientific Institute, Milan, Italy; 10 Department of Neuro Ophthalmology and Emergencies, Rothschild Foundation Hospital, Paris, France; 11 Centre Hospitalier National d’Ophtalmologie des Quinze Vingts, Paris, France; 12 Institut Catala de Retina, Barcelona, Spain; 13 CHU Angers - Hôpital Hôtel Dieu, Angers, France; 14 Departments of Neurology and Ophthalmology, Wills Eye Hospital and Thomas Jefferson University, Philadelphia, PA USA; 15 Massachusetts Eye and Ear Infirmary, Boston, MA USA; 16 Alkek Eye Center, Houston, TX USA; 17 Doheny Eye Center UCLA, Department of Ophthalmology David Geffen School of Medicine at UCLA, Doheny Eye Institute, Los Angeles, CA USA; 18 Department of Ophthalmology, University of Ottawa Eye, Ottawa, ON Canada; 19 GenSight Biologics, Paris, France; 20 Université de la Sorbonne, INSERM, CNRS, Institut de la Vision, 75012 Paris, France; 21 Fondation Ophtalmologique A. de Rothschild, 25-29 rue Manin, 75019 Paris, France; 22 Department of Ophthalmology, The University of Pittsburgh School of Medicine, Pittsburgh, PA USA; 23 CHNO des Quinze-Vingts, Institut Hospitalo-Universitaire FOReSIGHT, INSERM-DGOS CIC, 1423 Paris, France; 24 Emory University School of Medicine, Atlanta, GA USA; 25 Wills Eye Hospital and Sidney Kimmel Medical College of Thomas Jefferson University, Philadelphia, PA USA; 26 Doheny Eye Institute/UCLA School of Medicine, Los Angeles, CA USA; 27 Department of Ophthalmology, Institut Catala de Retina, Retina, Spain; 28 Department of Ophthalmology, University Vita-Salute, IRCCS Ospedale San Raffaele, Milan, Italy

**Keywords:** Optic nerve diseases, Epidemiology

## Abstract

**Background/objectives:**

REALITY is an international observational retrospective registry of LHON patients evaluating the visual course and outcome in Leber hereditary optic neuropathy (LHON).

**Subjects/methods:**

Demographics and visual function data were collected from medical charts of LHON patients with visual loss. The study was conducted in 11 study centres in the United States of America and Europe. The collection period extended from the presymptomatic stage to at least more than one year after onset of vision loss (chronic stage). A Locally Weighted Scatterplot Smoothing (LOWESS) local regression model was used to analyse the evolution of best-corrected visual acuity (BCVA) over time.

**Results:**

44 LHON patients were included; 27 (61%) carried the m.11778G>A *ND4* mutation, 8 (18%) carried the m.3460G>A *ND1* mutation, and 9 (20%) carried the m.14484T>C *ND6* mutation. Fourteen (32%) patients were under 18 years old at onset of vision loss and 5 (11%) were below the age of 12. The average duration of follow-up was 32.5 months after onset of symptoms. At the last observed measure, mean BCVA was 1.46 LogMAR in *ND4* patients, 1.52 LogMAR in *ND1* patients, and 0.97 LogMAR in *ND6* patients. The worst visual outcomes were reported in *ND4* patients aged at least 15 years old at onset, with a mean BCVA of 1.55 LogMAR and no tendency for spontaneous recovery. The LOESS modelling curve depicted a severe and permanent deterioration of BCVA.

**Conclusions:**

Amongst LHON patients with the three primary mtDNA mutations, adult patients with the m.11778G>A *ND4* mutation had the worst visual outcomes, consistent with prior reports.

## Introduction

Leber hereditary optic neuropathy (LHON) is an inherited optic neuropathy caused by mitochondrial DNA (mtDNA) mutations, which affect complex I subunits of the mitochondrial respiratory chain, impairing mitochondrial respiration and increasing production of reactive oxygen species [1–4]. Retinal ganglion cells (RGCs) are particularly vulnerable to mitochondrial dysfunction, which may lead to apoptotic cell death and axonal degeneration, ultimately resulting in optic atrophy [5, 6]. LHON affects ~1 in 30,000 to 1 in 50,000 people [4, 7, 8], with a peak age of onset between 15 and 35 years, and a large male predominance [3, 9–12]. LHON is characterised by bilateral painless central vision loss, typically sequential, with the fellow eye undergoing disease conversion weeks to a few months after onset in the first eye [13–15]. There is a well-documented incomplete penetrance, implying that the mtDNA mutations are necessary, but not sufficient to precipitate visual loss. Although there is considerable interfamilial variability, approximately 50% of men and 10% of women who carry one of the primary LHON mutations will experience visual loss during their lifetimes, highlighting also a higher male prevalence [8, 14, 16].

Three mtDNA point mutations account for about 90% of LHON cases: m.11778G>A in *ND4*, m.3460G>A in *ND1*, and m.14484T>C in *ND6* [3, 4]. The m.11778G>A mutation is the most common cause of LHON worldwide and it is known to be a severe mutation with less than 15% of patients experiencing some degree of visual recovery [17, 18]. Children have a better visual prognosis, especially when age of onset is before 12 years [10, 19]. Treatment options for LHON remain limited, with some improvement demonstrated in subgroups of LHON patients treated with idebenone [20–22], and some early promising results with intravitreal gene therapy [23–26].

The REALITY study is an international multicenter observational retrospective registry of LHON patients designed to evaluate the natural history of the three most common disease-causing mtDNA mutations and the factors influencing the visual outcome.

## Methods

### Study design

Eleven study centres in the US and Europe (France, Italy, Spain, and the United Kingdom) participated in the REALITY registry. To qualify for study inclusion, patients had to have a diagnosis of LHON confirmed by genotyping for one of the three primary mutations (m.11778G>A in *ND4*, m.3460G>A in *ND1* and m.14484T>C in *ND6*). Furthermore, patients were included only if they had undergone at least two visual function assessments, performed at any time between Year 1 and Year 3 after onset of vision loss. There was no restriction on age, and patients could have received idebenone or any other treatment. The primary source of demographic and clinical data was the enroled subjects’ medical records.

The relevant local Independent Ethics Committees approved the study protocol before subjects were identified and data collected. For patients under 18 years old, permission from a legal guardian was required to participate in the study. This study was conducted in accordance with the provisions of the Declaration of Helsinki and Good Clinical Practice guidelines.

### Best-corrected visual acuity (BCVA) analysis

On-chart BCVA measures, expressed in decimal fraction or Snellen notation, were converted into LogMAR values. Off-chart BCVA was assigned the following LogMAR values: 2.0 for count fingers; 2.3 for hand motion based on the Lange scale equivalence [27]; 4.0 for light perception; and 4.5 for no light perception.

### Data analysis

The Full Analysis Set included all enroled subjects whose eligibility was confirmed. Subgroup analyses were performed by LHON genotype (*ND4, ND1, ND6*), age at onset of vision loss (two cut-offs were applied: 12 and 15 years old), and idebenone treatment status. Change in BCVA was evaluated from the presymptomatic phase to the last available clinic observation. Missing data for presymptomatic BCVA were imputed a LogMAR value of 0 (Snellen equivalent 20/20; decimal fraction equivalent 1) [28, 29]. No other data imputation were performed for missing data.

A Locally Weighted Scatterplot Smoothing (LOWESS), non-parametric, local regression model was used on the individual BCVA data points of the LHON *ND4* patients who were at least 15 years old at onset. The resulting curve depicting the evolution of BCVA over time was based on a series of polynomial regressions around each data point. The regressions used a limited look back and look forward, giving distant points less weight. The starting point of the curve was set at the onset of vision loss and included presymptomatic values (missing presymptomatic data were imputed 0 LogMAR). All computations and generation of analysis datasets and tables were performed using SAS^®^ software version 9.4 or higher (SAS Institute, Cary, NC, USA).

## Results

### Study population characteristics and follow-up period

A total of 44 affected LHON patients were included in the REALITY study: 34/44 (77%) from European countries (France, Italy, Spain, and the United Kingdom) and 10/44 (23%) from the United States of America (Table 1). The majority of enroled patients were male (33/44, 75%) and Caucasian (33/44, 75%) (Table 1). The mean age at onset of vision loss was 27.9 years (range: 4–71 years), with 30/44 (68%) patients ≥18 years old at the onset of vision loss. The proportion of patients who were at least 15 years old at onset was 82% (36/44). Five (11%) patients were 12 years old or younger at onset.

**Table 1:** Demographics of LHON cohort.

	All patients (*N* = 44)	ND4 patients (*N* = 27)	ND1 patients (*N* = 8)	ND6 patients (*N* = 9)	ND4 patients aged ≥ 15 at onset (*N* = 23)
Age at onset of vision loss^a^ (years)					
Mean (SD)	27.9 (18.7)	30.2 (20.1)	24.9 (15.8)	23.6 (17.7)	34.2 (19.0)
Median	21.5	23.0	17.5	21.0	25.0
IQR	16.0, 32.0	16.0, 39.0	15.5, 29.0	14.0, 24.0	19.0, 57.0
Min, Max	4, 71	4, 71	14, 61	8, 68	16, 71
Categories of age at onset^a^					
≤12 years old	5/44 (11.4%)	3/27 (11.1%)	0/8 (0.0%)	2/9 (22.2%)	NA
13 to 14 years old	3/44 (6.8%)	1/27 (3.7%)	1/8 (12.5%)	1/9 (11.1%)	NA
15 to 17 years old	6/44 (13.6%)	3/27 (11.1%)	3/8 (37.5%)	0/9 (0.0%)	3/23 (13.0%)
18 and older	30/44 (68.2%)	20/27 (74.1%)	4/8 (50.0%)	6/9 (66.7%)	20/23 (87.0%)
Sex					
Male	33/44 (75.0%)	22/27 (81.5%)	5/8 (62.5%)	6/9 (66.7%)	18/23 (78.3%)
Female	11/44 (25.0%)	5/27 (18.5%)	3/8 (37.5%)	3/9 (33.3%)	5/23 (21.7%)
Ethnicity					
Caucasian/White	33/44 (75.0%)	20/27 (74.1%)	5/8 (62.5%)	8/9 (88.9%)	18/23 (78.3%)
Black	1/44 (2.3%)	1/27 (3.7%)	0/8 (0.0%)	0/9 (0.0%)	1/23 (4.3%)
Asian	1/44 (2.3%)	1/27 (3.7%)	0/8 (0.0%)	0/9 (0.0%)	0/23 (0.0%)
Unknown	9/44 (20.4%)	5/27 (18.5%)	3/8 (37.5%)	1/9 (11.1%)	4/23 (17.4%)
Country of origin					
France	9/44 (20.5%)	6/27 (22.2%)	3/8 (37.5%)	0/9 (0.0%)	5/23 (21.7%)
Italy	12/44 (27.3%)	6/27 (22.2%)	4/8 (50.0%)	2/9 (22.2%)	5/23 (21.7%)
Spain	5/44 (11.4%)	2/27 (7.4%)	1/8 (12.5%)	2/9 (22.2%)	2/23 (8.7%)
United Kingdom	8/44 (18.2%)	5/27 (18.5%)	0/8 (0.0%)	3/9 (33.3%)	4/23 (17.4%)
United States	10/44 (22.7%)	8/27 (29.6%)	0/8 (0.0%)	2/9 (22.2%)	7/23 (30.4%)

*IQR* inter-quartile range, *NA* not applicable, *SD* standard deviation.

^a^Onset in first-affected eye.

Three LHON patients had been diagnosed with an additional genetic syndrome: (i) the blepharophimosis, ptosis, and epicanthus inversus syndrome; (ii) the Ehlers–Danlos syndrome; (iii) and the Marcus–Gunn syndrome. In addition, one woman was diagnosed with multiple sclerosis following the onset of visual loss from LHON.

Most patients (27/44, 61%) were seen by an ophthalmologist or a neuro-ophthalmologist within the first 3 months of disease onset. The mean time from onset to the first visit was 5.7 months (range: 0–25.7 months), and the mean duration of follow-up was 32.5 months (range: 14.1–178.3 months) (Table 2). Seven patients (7/44, 16%) had a follow-up of more than 3 years, 6 of whom carried the m.11778G>A mutation and one the m.14484T>C mutation. The mean number of BCVA visits per patient was 7.9, with a range of 4–28 visits reported per patient.

**Table 2:** BCVA data collection in REALITY.

	All patients (*N* = 44)	*ND4* patients (*N* = 27)	*ND1* patients (*N* = 8)	*ND6* patients (*N* = 9)	*ND4* patients aged ≥ 15 at onset (*N* = 23)
Time from onset to first visit (months)					
Mean (SD)	5.7 (7.3)	6.9 (7.8)	3.0 (6.2)	4.5 (6.2)	6.8 (7.2)
Median	1.9	2.9	0.6	1.3	3.0
IQR	0.5, 11.2	0.8, 14.5	0.2, 2.0	1.1, 6.0	0.8, 14.5
Min, Max	0.0, 25.7	0.0, 25.7	0.0, 18.2	0.0, 17.8	0.0, 20.5
Time period of first visit post-onset					
≤1 month	14/44 (31.8%)	7/27 (25.9%)	5/8 (62.5%)	2/9 (22.2%)	6/23 (26.1%)
>1 to 3 months	13/44 (29.5%)	7/27 (25.9%)	2/8 (25.0%)	4/9 (44.4%)	5/23 (21.7%)
>3 to 6 months	4/44 (9.1%)	3/27 (11.1%)	0/8 (0.0%)	1/9 (11.1%)	3/23 (13.0%)
>6 to 12 months	4/44 (9.1%)	3/27 (11.1%)	0/8 (0.0%)	1/9 (11.1%)	3/23 (13.0%)
>2 months	9/44 (20.5%)	7/27 (25.9%)	1/8 (12.5%)	1/9 (11.1%)	6/23 (26.1%)
Time from onset to last visit (months)					
Mean (SD)	32.5 (24.1)	36.3 (29.8)	26.0 (6.0)	26.7 (8.6)	36.8 (32.2)
Median	30.3	30.4	27.3	30.3	29.3
IQR	23.3, 34.5	25.0, 35.4	22.2, 31.1	18.7, 35.1	24.4, 35.3
Min, Max	14.1, 178.2	15.9, 178.2	15.3, 31.4	14.1, 36.0	15.9, 178.2
Time period of last visit post-onset					
≤18 months	4/44 (9.1%)	1/27 (3.7%)	1/8 (12.5%)	2/9 (22.2%)	1/23 (4.3%)
>18 to 24 months	8/44 (18.2%)	4/27 (14.8%)	2/8 (25.0%)	2/9 (22.2%)	4/23 (17.4%)
>24 to 30 months	9/44 (20.5%)	8/27 (29.6%0	1/8 (12.5%)	0/9 (0.0%)	7/23 (30.4%)
>30 to 36 months	16/44 (36.4%)	8/27 (29.6%)	4/8 (50.0%)	4/9 (44.4%)	6/23 (26.1%)
>36 months	7/44 (15.9%)	6/27 (22.2%)	0/8 (0.0%)	1/9 (11.1%)	5/23 (21.7%)
Number of eyes with BCVA data available in time period					
Up to Year 1 after onset	59/88 (67.0%)	35/54 (64.8%)	10/16 (62.5%)	14/18 (77.8%)	31/46 (67.4%)
Year 1 to Year 2 after onset	76/88 (86.4%)	44/54 (81.5%)	16/16 (100.0%)	16/18 (88.9%)	39/46 (84.8%)
Year 2 to Year 3 after onset	59/88 (67.0%)	42/54 (77.8%)	10/16 (62.5%)	7/18 (38.9%)	34/46 (73.9%)
>Year 3 after onset	14/88 (15.9%)	12/54 (22.2%)	0/16 (0.0%)	2/18 (11.1%)	10/46 (21.7%)
Number of BCVA visits per patient					
Mean (SD)	7.9 (4.9)	7.2 (3.9)	8.0 (3.7)	9.9 (8.0)	7.3 (4.2)
Median	6.0	6.0	7.0	6.0	6.0
IQR	5.5, 8.0	5.0, 8.0	6.0, 9.0	6.0, 10.0	4.0, 8.0
Min, Max	4, 28	4, 22	4, 16	4, 28	4, 22

*BCVA* best-corrected visual acuity, *IQR* interquartile range, *SD* standard deviation.

Date of onset of vision loss in the first eye to be affected was used to calculate times from onset for each patient.

### LHON genotypes

Twenty-seven patients (27/44, 61%) carried the m.11778G>A mutation in *ND4*, 8 patients (8/44, 18%) carried the m.3460G>A mutation in *ND1*, and 9 patients (9/44, 21%) carried the m.14484T>C mutation in *ND6*, with a mean age at onset of 30.2, 24.9, and 23.6 years, respectively (Table 1). Among the 36 patients who were at least 15 years old at onset, there were 23 *ND4* patients (64%), 7 *ND1* patients (19%) and 6 *ND6* patients (17%).

### Idebenone status

Twenty-five patients (25/44, 57%) had taken idebenone with a mean cumulative duration of treatment of 55.7 months (Supplementary Table S1). The treated group included 16/27 (59%) *ND4* patients, 5/8 (62%) *ND1* patients and 4/9 (44%) *ND6* patients. Treatment was started during the first year after disease onset in 21/25 (84%) patients.

### BCVA evolution based on LHON genotypes and age of onset

For the entire study cohort, the mean (standard deviation (SD)) BCVA was 1.37 (0.78) LogMAR at last observed measure (mean of 32.2 months from onset of vision loss) (Table 3). Based on the LHON genotype, the mean (SD) BCVA was 1.46 (0.63) LogMAR in *ND4* patients, 1.52 (1.06) LogMAR in *ND1* patients, and 0.97 (0.83) LogMAR in *ND6* patients (Table 3).

**Table 3:** BCVA outcomes according to LHON genotypes.

	All patients (*N* = 88 eyes)	*ND4* patients (*N* = 54 eyes)	*ND1* patients (*N* = 16 eyes)	*ND6* patients (*N* = 18 eyes)	*ND4* patients aged ≥ 15 at onset (*N* = 46 eyes)	*ND4* patients aged ≥ 15 at onset and treated with idebenone (*N* = 30 eyes)
Time from onset to last visit (months)						
Mean (SD)	32.2 (24.0)	36.3 (29.5)	26.0 (5.8)	25.6 (8.6)	36.8 (31.8)	40.5 (38.0)
Presymptomatic BCVA (LogMAR)^a^	0	0	0	0	0	0
Last-observed BCVA (LogMAR)						
Mean (SD)	1.37 (0.78)	1.46 (0.63)	1.52 (1.06)	0.97 (0.83)	1.55 (0.62)	1.57 (0.62)
95% CI	1.15, 1.59	1.17, 1.74	1.00, 2.04	0.48, 1.46	0.96, 1.54	0.71, 1.52
Median	1.30	1.47	1.60	0.75	1.60	1.60
Minimum, maximum	0.00, 4.00	0.00, 2.30	0.10, 4.00	0.00, 2.30	0.00, 2.30	0.10, 2.30
Q1, Q3	0.80, 2.00	1.10, 2.00	0.00, 2.00	0.22, 2.00	1.20, 2.00	1.10, 2.00

*BCVA* best-corrected visual acuity; *SD* standard deviation.

^a^Missing presymptomatic BCVA were assigned a value of 0 LogMAR (normal visual acuity).

At last observed measure, LHON patients ≤ 12 years old at onset had a mean (SD) BCVA of 0.65 (0.52) LogMAR, compared with 1.46 (0.77) LogMAR in patients over 12 years old at onset (*p* = 0.0193) (Supplementary Table S2).

**Fig. 1:**
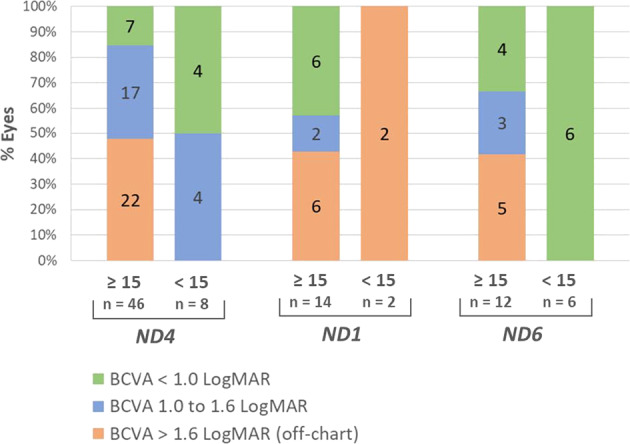
BCVA data collection in REALITY. The distribution of BCVA data collected in the REALITY registry is presented according to LHON genotype and age at onset (below 15 or at least 15 years old). The number of eyes is displayed for each category.

When considering LHON patients aged at least 15 years at onset, 55/72 (76%) of eyes had a BCVA of 1.0 LogMAR or worse at last observed measure: 39/46 (85%) for *ND4* patients, 8/14 (57%) for *ND1* patients and 8/12 (67%) for *ND6* patients (Fig. 1). The proportion of eyes with off-chart BCVA was comparable for the three LHON genotypes: 22/46 (48%) for *ND4* patients, 6/14 (43%) for *ND1* patients and 5/12 (42%) for *ND6* patients. The proportion of patients with a BCVA better than 1.0 LogMAR in at least one eye at the last observed measure was 5/23 (22%) for *ND4* patients; 3/7 (43%) for *ND1* patients and 2/6 (33%) for *ND6* patients.

For LHON patients who were below 15 years of age at onset, 6/16 (37%) eyes had a BCVA of 1.0 LogMAR or worse at last observed measure: 4/8 (50%) of *ND4* patients, 2/2 (100%) of *ND1* patients and 0/6 (0%) of *ND6* patients (Fig. 1). Of note, no eyes were off-chart at the last observed measure in *ND4* and *ND6* patients who were less than 15 years old at onset (Fig. 1).

### Subgroup analysis of LHON *ND4* patients aged ≥15 years at onset

Twenty-three LHON patients carrying the m.11778G>A *ND4* mutation were at least 15 years old at onset, with the majority being male (18/23, 78.3%). The mean (SD) age at onset was 34.2 (19.0) years and 3/23 (13%) patients were between 15 and 18 years old at onset of vision loss (Table 1). The mean (SD) time from onset to the first visit was 6.8 (7.2) months and the mean duration of follow-up was 36.8 (32.2) months for this subgroup (Table 2).

The mean (SD) BCVA was 1.55 (0.62) LogMAR at the last observed measure (Table 3). Fifteen (15/23, 65%) *ND4* patients aged at least 15 years at onset were treated with idebenone during the observational period (Supplementary Table S1). For nine of these patients, the mean (SD) cumulative duration of treatment with idebenone was 70.0 (24.9) months. At the last observed measure, the mean (SD) BCVA for the idebenone-treated group was 1.57 (0.62) LogMAR on average 40.5 (38.0) months after onset (Table 3).

**Fig. 2:**
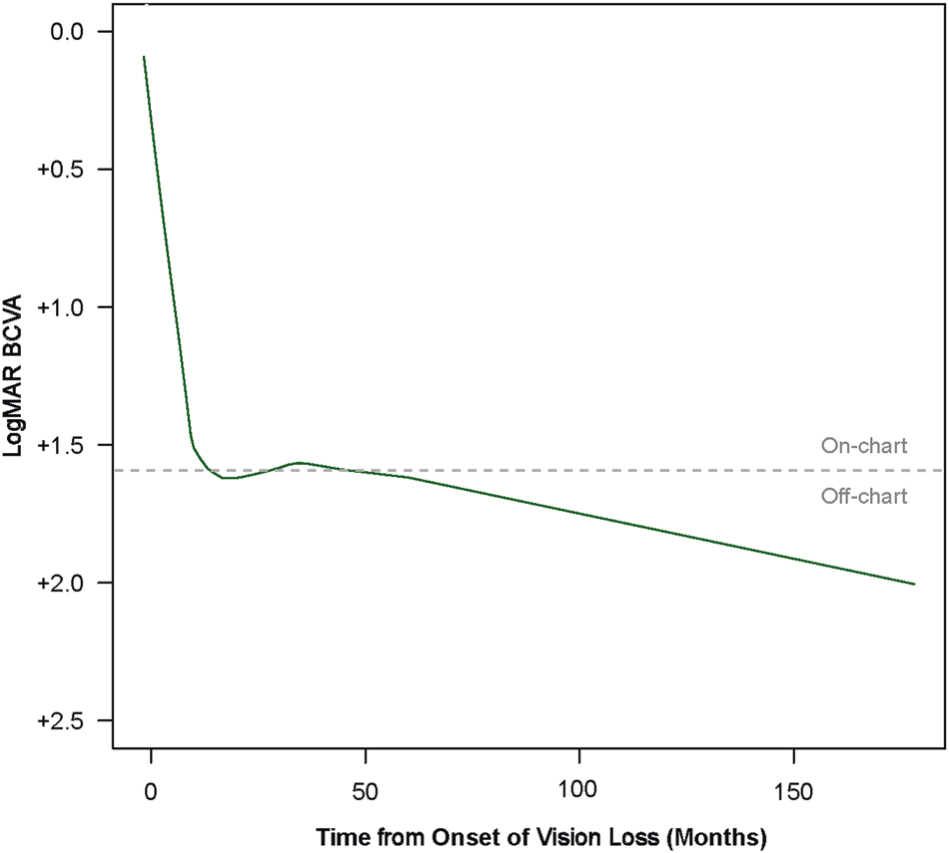
BCVA evolution in *ND4*patients aged ≥15 at onset of vision loss. The LOWESS regression curve for *ND4* patients aged at least 15 at onset of vision loss (*n* = 23 patients, 213 individual BCVA measures) was based on fitting a series of linear regressions around each data point collected. The regressions used a limited look back and look forward, giving distant points less weight. The starting point of the curves was set at the onset of vision loss and included presymptomatic values (missing presymptomatic data were imputed a value of 0 LogMAR).

The LOWESS curve depicting the evolution of BCVA over time showed a marked loss of visual acuity in the first 18 months after disease onset (Fig. 2). Following this initial drop of vision, the deterioration of BCVA continued at a slower rate and no trend of visual recovery was observed.

## Discussion

The REALITY study cohort is representative of the general LHON patient population with regards to the age of onset (mean of 27.9 years) and gender distribution (75% males) [3, 12]. Prior studies have estimated the peak age of onset to be between 15 and 35 years old, although the reported range for age at onset in molecularly-confirmed LHON patients varies from 2 to 87 years of age [3, 10, 11]. The proportion of patients in REALITY who were less than 15 years old at disease onset was 18% (8/44) and the predominant genotype among all included patients was the m.11778G>A *ND4* mutation (61%), also in keeping with the literature. [5, 6, 15, 17, 30–32] Half of the patients enroled in REALITY had received treatment with idebenone for a cumulative period of more than 4 years.

The genetic syndromes reported in three patients included in REALITY are unlikely to be related to the underlying LHON-causative mtDNA mutation. One additional patient was diagnosed with multiple sclerosis. The co-occurrence of visual loss due to LHON and central nervous system demyelination is often referred to as Harding’s disease [33], and this overlap syndrome can occur with all three primary LHON mutations, predominantly in women [15, 33, 34].

The two most important predictors of visual outcome in LHON are the underlying causative mtDNA mutation and the age at onset of vision loss. In the REALITY study, LHON patients carrying the m.14484T>C mutation achieved better final vision compared with those carrying the m.11778G>A and m.3460G>A mutations. The literature reports that children who become affected at the age of 12 years or younger achieve significantly better vision compared with adult-onset LHON patients [13, 17, 19, 35]. Accordingly, in the REALITY registry we observed that patients ≤12 years old at onset had a mean final BCVA of 0.65 LogMAR, compared with 1.46 LogMAR for those over 12 years old at onset. Furthermore, the majority of eyes of LHON patients who were below the age of 15 at onset had a BCVA better than 1.0 LogMAR at the last observed measure, and all the *ND4* and *ND6* patients younger than 15 at onset had on-chart visual acuity in both eyes, confirming the better visual prognosis in these younger groups.

In the LHON *ND4* subgroup of patients who were at least 15 years old at onset, the majority of eyes had a BCVA of 1.0 LogMAR or worse at the last observed measure. The m.11778G>A mutation is known to carry a poor prognosis, with <15% of patients experiencing spontaneous partial visual recovery, and few achieving visual acuities better than 20/200. [17] In REALITY, 84.8% (39/46) of eyes from LHON *ND4* patients who were at least 15 at onset had BCVA of 20/200 or worse, and 47.8% (22/46) were off-chart at the last observed measure. In this subgroup, a LOWESS model curve showed a decline in two phases: a rapid decline in the first 18 months, followed by a progressive slow decline. A prospective long-term follow-up study of untreated LHON *ND4* patients is needed to confirm the projection of this model, which has important implications for the treatment of chronic cases.

The REALITY registry bears a number of limitations due to the retrospective nature of its study design and the relatively small sample size of 44 LHON patients. Nevertheless, the findings of this study confirm the better visual outcome with childhood-onset LHON and the poor visual prognosis for patients with the m.11778G>A mutation who were at least 15 years old at disease onset.

### Summary

#### What was known before

- LHON is a rare mitochondrial blinding disease affecting mostly males between 15 and 35 years old.
- Three primary mtDNA mutations are responsible for 90% of the cases.
- Spontaneous partial visual recovery is scarcely reported in the literature.

#### What this study adds

- This international retrospective registry study provided a better understanding of the evolution of visual acuity in LHON patients, including clinical data from 44 subjects aged 4 to 71 years at onset of vision loss.
- LHON subjects carrying the *ND4* mutation did not show a trend for spontaneous improvement over time.
- LHON subjects aged 12 or less at onset showed the best visual prognosis.

## Supplementary information

S1

S2

## Data Availability

All data associated with this study are available in the main text or the supplementary materials
