# Supplementary material for: Natural history of patients with Leber hereditary optic neuropathy—results from the REALITY study
Source: Eye (Lond). 2021 Apr 28;36(4):818–26. doi: 10.1038/s41433-021-01535-9 (PMC8956580; doi:10.1038/s41433-021-01535-9)
Supplement: Supplementary file 1 — S1 [file 41433_2021_1535_MOESM1_ESM.docx]

Supplementary Table S1. Timing and duration of treatment with idebenone

|  | **All patients**  **(N = 44)** | ***ND4* patients**  **(N = 27)** | ***ND1* patients**  **(N = 8)** | ***ND6* patients**  **(N = 9)** | ***ND4* patients**  **aged ≥ 15 at onset**  **(N = 23)** | |
| --- | --- | --- | --- | --- | --- | --- |
| **LHON patients treated with idebenone** | |  |  |  |  | |
| Treatment started ≤ 12 months after onset | 21 / 44 (47.7%) | 13 / 27 (48.1%) | 5 / 8 (62.5%) | 3 / 9 (33.3%) | 12 / 23 (52.2%) | |
| Treatment started > 12 months after onset | 4 / 44 (9.1%) | 3 / 27 (11.1%) | 0 / 8 (0.0%) | 1 / 9 (11.1%) | 3 / 23 (13.0%) | |
| Not treated | 19 / 44 (43.2%) | 11 / 27 (40.7%) | 3 / 8 (37.5%) | 5 / 9 (55.6%) | 8 / 23 (34.8%) | |
| **Cumulative duration of treatment with idebenone (months)** | | | | | |  |
| Patients with available data | 15 | 10 | 5 | 0 | 9 | |
| Mean (SD) | 55.7 (28.6) | 68.6 (23.9) | 30.0 (18.4) | - | 70.0 (24.9) | |
| Median | 56.0 | 64.5 | 25.0 | - | 66.0 | |
| IQR | 28.0, 68.0 | 55.0, 87.0 | 24.0, 28.0 | - | 55.0, 87.0 | |
| Min, Max | 12, 107 | 31, 107 | 12, 61 | - | 31, 107 | |
| Patients with missing data | 10 | 6 | 0 | 4 | 6 | |
