# Supplementary material for: Natural history of patients with Leber hereditary optic neuropathy—results from the REALITY study
Source: Eye (Lond). 2021 Apr 28;36(4):818–26. doi: 10.1038/s41433-021-01535-9 (PMC8956580; doi:10.1038/s41433-021-01535-9)
Supplement: Supplementary file 2 — S2 [file 41433_2021_1535_MOESM2_ESM.docx]

Supplementary Table 2. BCVA outcomes according to age at onset

|  | **All patients**  **(N = 88 eyes)** | **Aged ≤12 at onset**  **(N = 10 eyes)** | **Aged >12 at onset**  **(N = 78 eyes)** |
| --- | --- | --- | --- |
| **Time from onset to last visit (months)** | | | |
| Mean (SD) | 32.2 (24.0) | 29.1 (6.9) | 32.6 (25.4) |
| **Presymptomatic BCVA (LogMAR) ^a^** | 0 | 0 | 0 |
| **Last‑observed BCVA (LogMAR)** | | | |
| Mean (SD) | 1.37 (0.78) | 0.65 (0.52) | 1.46 (0.77) |
| 95% CI | 1.15, 1.59 | 0.02, 1.28 | 1.23, 1.69 |
| Median | 1.30 | 0.70 | 1.51 |
| Minimum, maximum | 0.00, 4.00 | 0.00, 1.40 | 0.00, 4.00 |
| Q1, Q3 | 0.80, 2.00 | 0.00, 1.10 | 0.90, 2.00 |
| Repeated-measures ANOVA P value ^b^ | | *0.0193* | |
| a: missing presymptomatic BCVA were assigned a value of 0 LogMAR (i.e. normal visual acuity).  b: Comparison of mean BCVA between patients ≤ 12 years old and > 12 years old at onset. | | | |
